# Microbial Protein and Metabolite Profiles of *Klebsiella oxytoca M5A1* in a Bubble Column Bioreactor

**DOI:** 10.3390/biotech13040043

**Published:** 2024-10-19

**Authors:** Tawakalt Ayodele, Musiliu Liadi, Abodunrin Tirmidhi Tijani, Kudirat Alarape, Christiana Bitrus, Clairmont L. Clementson, Ademola Hammed

**Affiliations:** 1 Environmental and Conservation Sciences, North Dakota State University, Fargo, ND 58102, USA; tawakalt.ayodele@ndsu.edu (T.A.); abodunrin.tijani@ndsu.edu (A.T.T.);; 2 Agricultural and Biosystems Engineering, North Dakota State University, Fargo, ND 58108, USA; clairmont.clementson@ndsu.edu

**Keywords:** microbial protein, bubble column bioreactor, static fermentation, *Klebsiella oxytoca M5A1*, organic acids

## Abstract

The production of microbial proteins (MPs) has emerged as a critical focus in biotechnology, driven by the need for sustainable and scalable alternatives to traditional protein sources. This study investigates the efficacy of two experimental setups in producing MPs using the nitrogen-fixing bacterium *Klebsiella oxytoca M5A1*. *K. oxytoca M5A1*, known for its facultative anaerobic growth and capability to fix atmospheric nitrogen, offers a promising avenue for environmentally friendly protein production. This research compares the performance of a simple bubble column (BC) bioreactor, which promotes efficient mixing and cross-membrane gas transfer, with static fermentation, a traditional method lacking agitation and aeration. The study involved the parallel cultivation of *K. oxytoca M5A1* in both systems, with key parameters such as microbial growth, glucose utilization, protein concentration, and metabolite profiles monitored over a 48 h period. The results indicate that the BC bioreactor consistently outperformed static fermentation regarding the growth rate, protein yield, and glucose utilization efficiency. The BC exhibited a significant increase in protein production, reaching 299.90 µg/mL at 48 h, compared to 219.44 µg/mL in static fermentation. The organic acid profile reveals both synthesis and utilization regimes of varying patterns. These findings highlight the advantages of the BC bioreactor for MP production, particularly its ability to maintain aerobic conditions that support higher growth and yield.

## 1. Introduction

MP production is a key process in the biotechnology industry, producing high-protein biomass rich in essential amino acids, vitamins, minerals, and other nutrients [1]. MPs can be produced efficiently in bioreactors, offering stable growth conditions, precise nutrient utilization, minimal water and land usage, and the elimination of the need for pesticides or antibiotics [2]. This method contrasts with traditional agriculture, which often results in nutrient waste and environmental issues such as aquifer contamination, the eutrophication of water bodies, ocean acidification, and greenhouse gas emissions. When produced in bioreactors, nearly all of the supplied nutrients are converted into consumable protein with minimal environmental impact [2]. Bioreactors are closed systems in which a biological process like fermentation can be carried out under controlled environmental conditions. It is a system in which a substrate is utilized by living cells to generate a product of higher value [3].

The success of these processes depends on the availability and accessibility of nutrients to the microbes. Microbial growth and product formation in fermentation systems are driven by the uptake of nutrients from the medium, which fuels the cellular metabolism and biosynthesis [4]. However, there exists a significant phase barrier in the nutrient uptake process, in which gases, including those essential for metabolic processes like nitrogen fixation, face difficulties in crossing the microbe membrane directly. The membrane functions as a semi-permeable system, regulating the absorption of nutrients based on the size, polarity, and solubility of the molecule [5]. While it facilitates the passage of certain molecules such as glucose [6], it presents challenges for others, particularly gases due to their low density, diffusivity, and solubility.

Gases, such as oxygen, nitrogen, or carbon dioxide, are non-polar molecules and can pass through the lipid bilayer of the bacterial membrane by diffusion. However, their entry is often limited by the gas solubility and concentration gradient [7]. In BC reactors, gases are introduced into the liquid medium, where they disperse into small bubbles. For these gases to be absorbed by bacteria, they must first dissolve in the liquid. The small size of the bubbles increases the surface area available for gas exchange between the gas phase and the liquid phase [4,8]. This circulation promotes mixing and helps to break the gas–liquid barrier by continuously bringing fresh liquid into contact with the gas bubbles. However, if the gas solubility is low, the transfer of gases from the medium to the bacteria becomes inefficient [9]. For example, both oxygen and nitrogen have low solubility in liquid, which can limit the availability of these gases for bacterial uptake. In nitrogen-fixing bacteria such as *Klebsiella oxytoca M5A1*, limited nitrogen can hinder the nitrogen fixation process [10]. Our research is founded on the principle that increasing the concentration of nitrogen-rich gases (>70%) in the bioreactor environment will enhance the availability of dissolved nitrogen in the medium. This approach aims to facilitate the uptake of nitrogen, which is crucial for nitrogen fixation processes.

This research leverages the nitrogen-fixing ability and facultative anaerobic nature of *K. oxytoca M5A1* for MP and metabolite production. *K. oxytoca M5A1* is considered nonpathogenic due to its reduced production of capsules, fimbriae, and lipopolysaccharides compared to pathogenic *Klebsiella* strains [11,12]. These attributes minimize biosafety concerns while maintaining *K. oxytoca M5A1*’s potential for industrial applications [12].

In the BC bioreactor, the cultivation process involves the controlled displacement of nutrients, with improved mixing and cross-membrane gas transfer within the bioreactor. This method has been gaining attention for its potential to enhance protein production [13] by maintaining optimal bacterial growth and activity conditions. BC bioreactors are characterized by the continuous upward flow of gas bubbles through the liquid phase, promoting effective gas–liquid mixing and mass transfer. Unlike other types of BC reactors such as airlift bioreactors, they do not have distinct upflowing and downflowing regions [14]; instead, the gas bubbles rise uniformly, creating turbulence that enhances gas transfer and nutrient distribution [15]. These reactors are widely used in various chemical and biochemical processes, including wastewater treatment and bioprocessing applications, due to their simplicity and efficient operation.

In contrast, static fermentation occurs without agitation or the use of gas, a traditional method used in many protein production processes. Recent advancements in bioreactor design and technology have raised questions about the efficiency of static fermentation compared to techniques like the BC bioreactor [16]. Therefore, comparing these two bioreactor systems is particularly relevant for identifying the most efficient way to optimize microbial cultivation processes to increase product yields while minimizing nutrient consumption.

To achieve this goal, we cultured *K. oxytoca M5A1* in a BC bioreactor, using static fermentation as the control. This comprehensive approach will allow us to elucidate the distinct advantages and limitations of each system, contributing to the broader understanding of microbial fermentation dynamics and their practical implications for sustainable protein production. Key parameters such as the protein concentration, metabolite profiles, and biomass yield, and the overall process efficiency were meticulously monitored and analyzed. By systematically evaluating the performance of *K. oxytoca M5A1* in different cultivation environments, this research aims to advance the field of microbial biotechnology and support the development of more efficient and scalable production methods. The findings are expected to have significant implications for industries focused on sustainable protein sources, bioprocess optimization, reactor designs, and the utilization of facultative anaerobic bacteria in diverse biotechnological applications.

## 2. Methodology

### 2.1. Media and Growth Conditions

A fresh colony of *K. oxytoca M5A1* was sub-cultured onto Luria–Bertani agar containing tryptone (5 g), agar (7.5 g), yeast extract (2.5 g), and NaCl (5 g) in 500 mL of distilled water. An overnight culture of the *K. oxytoca M5A1* strain was prepared by inoculating fresh colonies into Super Optimal Broth (SOB) containing MgSO_4_ (2.4 g), NaCl (0.5 g), yeast extract (5 g), Peptone (20 g), and KCl (0.186 g) in 1 L of distilled water. The overnight culture was incubated for 24 h in an incubating shaker at 37 °C and 200 rpm (Benchtop Lab Systems, Geno Tech Inc., St. Louis, MO, USA). The cultivation broth was prepared with minimal medium containing Na_2_HPO_4_·7H_2_O (25 g), CaCl_2_–2H_2_O (0.1 g), Na_2_MoO_4_·2H_2_O (0.25 mg), MgSO_4_·7H_2_O (0.25 g), KH_2_PO_4_ (3 g), FeCl_2_·4H_2_O (2.9 g), and NaCl (1 g) in 1 L of distilled water and a 10 g/L glucose solution. All chemicals, including the reagents used for the preparation of the SOB and minimal media, were laboratory grade and obtained from Sigma-Aldrich (St. Louis, MO, USA).

### 2.2. Fabrication of BC Bioreactor

**Figure 1:**
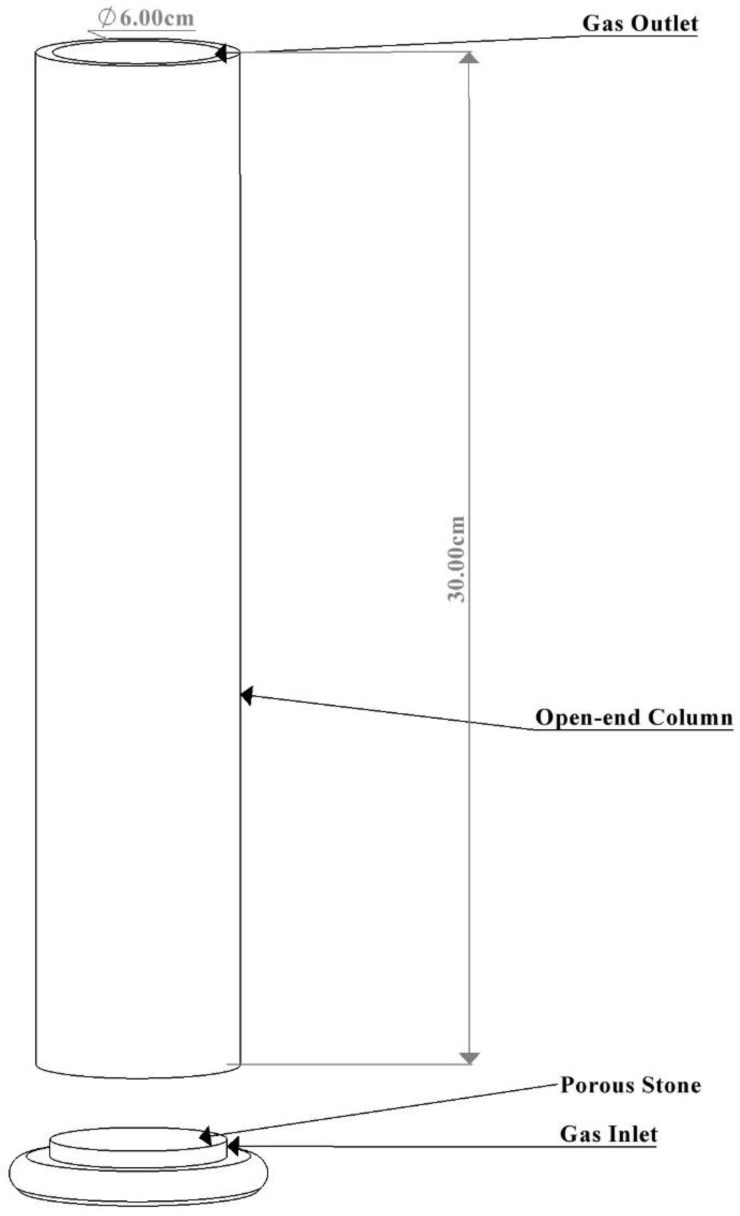
Fabricated bubble column bioreactor.

An open-end column (approximately 6 cm × 30 cm) capable of containing 250 mL of liquid media was used for the BC bioreactor construction. A porous stone with a gas inlet was incorporated and sealed into the column. The assembled bioreactor was left undisturbed for 24 h to ensure the proper adhesion of all components before use. Following this period, the bioreactor was sterilized with 70% ethanol and covered with sterile cotton to prevent contamination. A gas pipe was attached to the gas inlet of the BC bioreactor, and an air filter was positioned along the gas pipe to ensure only sterile gases entered the column, preventing contamination during cultivation. The schematic diagram of the BC bioreactor is presented in Figure 1 below.

### 2.3. Experimental Setup and Cultivation Conditions

For cultivation, the sucrose solution was prepared separately, sterilized by autoclaving for 20 min, and then allowed to cool in a water bath (Thermo Scientific MaxQ 7000, Waltham, MA, USA). Once cooled, the minimal media was supplemented with the sucrose solution in 1:1 *v*/*v* (pH of 6.5–7.5) and transferred separately into the fabricated BC bioreactor (6 cm × 30 cm) and Ermenleyer flasks for static fermentation (control). The minimal media (150 mL each) was inoculated with the overnight culture (5%) and incubated for 48 h at 37 °C in a water bath (Thermo Scientific MaxQ 7000, Waltham, MA, USA). Both setups were subjected to the same conditions, and had the same media volume and inoculation rate. Additionally, the BC was aerated (consisting mainly of 78% nitrogen gas and 21% oxygen) with a flow rate of 1.5 L/min. The samples were collected at 12 h intervals (0, 12, 24, and 48 h). The samples were plated on LB agar and incubated at 37 °C to observe colony morphology and to check for any contamination. The optical density (OD600) of each collected sample was measured to assess the microbial growth using a microplate spectrophotometer (Tecan, Männedorf, Switzerland).

### 2.4. Analytical Methods: Glucose Utilization and Organic Acid Production

Glucose utilization and organic acids (propionic acid, lactic acid, and acetic acid) were analyzed and quantified with a high-performance liquid chromatography system using LabSolutions Software, version 5.57 (Shimadzu LC-2050C HPLC, Tokyo, Japan) [17]. The mobile phase consisted of 0.01 N sulfuric acid at a flow rate of 1.0 mL/min. Prior to injection into the column, the samples were filtered using a 0.45 µm nylon filter. Microcentrifuge tubes and 2 mL autosampler vials were prepared and labeled with the respective sample identifiers. Each sample container was inverted to ensure proper mixing, and 2 mL of the resulting homogeneous solution was transferred to the labeled microcentrifuge tube. The sample aliquots were centrifuged for a minimum of 3.5 min at 10,000 rpm. The supernatants were subsequently filtered through a 1 cc syringe attached to a 0.45 µm nylon filter into the 2 mL autosampler vials. The results were first organized and calculated using Microsoft Excel 2016, and further statistical analysis was performed using Minitab software (version 21), with concentrations reported in g/L.

### 2.5. Protein Quantification

The protein concentration was determined using the Bradford assay protocol at each time point. A standard curve was established using Bovine Serum Albumin (BSA) for the assay [18]. A sample was pipetted into a test tube, and Bradford reagent was added at a ratio of 1:5 (*v*/*v*) [17]. The mixture was then thoroughly mixed and incubated at room temperature for 20 min, after which the absorbance was measured at a wavelength of 595 nm using the Tecan Infinite M Nano microplate reader (Tecan, Männedorf, Switzerland).

### 2.6. Statistical Analysis

Statistical analyses were conducted using Minitab statistical software (Version 21), which included a two-way Analysis of Variance (ANOVA) and a two-sample *t*-test to compare organic acid concentrations at each time point. The findings were presented as the mean ± standard deviation of three replicates.

## 3. Results and Discussion

### 3.1. Microbial Growth

**Figure 2:**
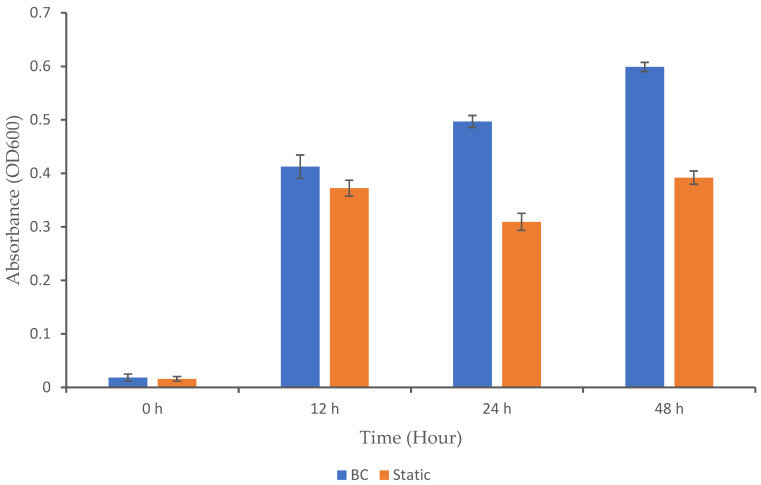
Growth profile of *K. oxytoca M5A1* under BC and static conditions. The data are presented with absorbance mean values ± SD of n = 3 experiments.

Microbial growth is influenced by several factors, including the type of bioreactor used and the bacterial strain involved. Different bioreactor designs have varying capabilities for gas transfer. In a BC bioreactor, gas is introduced at the bottom, forming bubbles that rise through the liquid and promote overall mixing and improved gas distribution [19]. However, for microorganisms like *K. oxytoca M5A1*, excessive oxygen can inhibit nitrogenase activity, which is crucial for nitrogen fixation [20]. In this case, the reactor must be carefully managed to maintain a balance between providing enough oxygen for respiration without inhibiting nitrogenase activity. To assess the impact of the BC bioreactor on microbial growth, we analyzed the microbial growth profile to determine whether this system positively or negatively affected the growth of *K. oxytoca M5A1* under the given conditions (Figure 2). The cultivation process began with the inoculation of an overnight culture of the *K. oxytoca M5A1* strain into the medium in both the BC and static (control) setups. To facilitate growth, we incubated the culture for 48 h at 37 °C. No contaminating microorganisms were detected, as only colonies consistent with the expected morphological characteristics of *K. oxytoca* M5A1 were observed during the cultivation. This observation confirms that the culture remained uncontaminated throughout the experiment.

In our study, the BC exhibited significantly higher growth (OD600) over time compared to the static condition (*p* < 0.05), with notable increases at 12, 24, and 48 h. Both setups exhibited low values at the initial hour. By 12 h, the BC showed a significant increase in growth from 0.02 to 0.41 (OD600), while the static setup exhibited a modest rise from 0.02 to 0.37. The BC continued its upward trajectory until 48 h, while the static setup experienced a slight decrease in growth at 24 h and formed a plateau at 48 h. The continuous upward growth trend in the BC system shows that the bioreactor maintained a balanced environment. A similar growth pattern in BC bioreactors has been observed in previous studies. For instance, a study reported that *Trichosporon* yeast are able to grow better in a BC reactor compared to stirred-tank bioreactor due to a stable physical environment [21]. Nadeem [22] also reported that *Rhizopus oligosporous* can grow when cultured in a BC bioreactor. Additionally, Zheng and colleagues documented the use of airlift bioreactors (a type of BC reactor) for culturing *Candida arborea* to produce microbial biomass [8]. Yen and Chang (2015) also reported that using an airlift bioreactor achieved higher cell mass growth as compared to the agitation tank [23].

The observed microbial growth in the BC can be attributed to its continuous mixing and aeration mechanisms. Aeration provides a consistent supply of essential gases, such as nitrogen and oxygen, which support both the respiration and the nitrogen-fixing ability of facultative microbes like *K. oxytoca M5A1*. Oxygen is critical for cellular respiration, while nitrogen is required for nitrogen fixation [10], a process facilitated by the nitrogenase enzyme. Continuous mixing, however, prevents the accumulation of toxic metabolic byproducts around the cells and ensures that the gases and nutrients in the medium, such as carbon sources and trace elements, are evenly distributed. This prevents the formation of nutrient concentration gradients that could otherwise lead to localized nutrient depletion and microbial starvation. The uniform distribution of nutrients allows all microbial cells to have access to the resources they need for growth and metabolism. As a result, the combination of aeration and mixing creates a stable and well-balanced environment that promotes robust microbial growth. The significant increase in growth over time in the BC, compared to the plateau observed in the static method, suggests that the BC may offer advantages in terms of the cultivation efficiency.

### 3.2. Glucose Utilization

**Figure 3:**
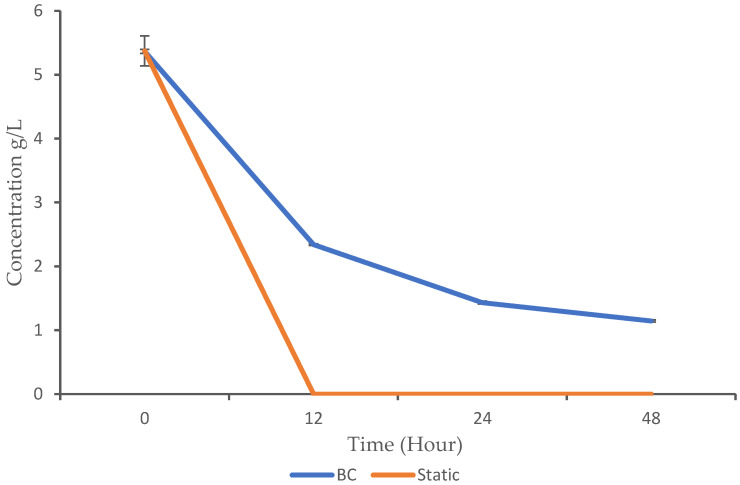
Glucose utilization pattern of *K. oxytoca M5A1* under the BC and static conditions. The data are presented with absorbance mean values ± SD of n = 3 experiments.

Microorganisms can metabolize glucose either aerobically or anaerobically. Aerobic metabolism requires oxygen and typically leads to the complete oxidation of glucose, producing energy (ATP) and reducing equivalents (NADH and FADH_2_) used for biosynthesis. The anaerobic metabolism occurs without oxygen and often involves the partial oxidation of glucose, producing less energy but allowing for growth under conditions where oxygen is scarce. In the context of this study, the glucose utilization profile was monitored using HPLC analysis. The BC demonstrated gradual glucose consumption over time, with a significant decrease from 5.36 g/L at 0 h to 1.14 g/L at 48 h. In contrast, static fermentation exhibited rapid glucose utilization, as it dropped to 0 g/L by 12 h (Figure 3).

The BC showed a gradual decrease in glucose levels, while glucose was completely depleted in the static condition after 12 h. The *p*-values from 12 h to 48 h are highly significant, which indicate a significant difference in glucose consumption between the BC and static conditions (*p*-value < 0.05). Despite the higher growth observed in the BC compared to the static fermentation, the microbes consumed less substrate. This raises the question of whether aeration and continuous mixing influence glucose consumption. While the specific comparison we conducted in this study is not widely reported, related findings show similar patterns. For instance, Zheng et al. [8] observed gradual glucose consumption by *Candida arborea* AS1.257 in airlift bioreactor. In addition, a report by [24] indicated that *Rhizopogon nigrescens* exhibited gradual glucose consumption when cultured in an airlift bioreactor. These findings support the idea that aeration and mixing can reduce glucose consumption rates in BC bioreactors. Similarly to the trend observed in our study, where the complete utilization of glucose occurred at 12 h in static fermentation, Ayodele et al. [17] also reported that *K. oxytoca M5A1* completely utilized glucose within 16 h when cultured under anaerobic agitated conditions.

The efficiency of glucose utilization during cultivation is crucial for determining the productivity and sustainability of fermentation processes. The BC supports reduced glucose utilization due to aeration, which promotes oxygen availability and facilitates the aerobic metabolism of glucose. This shifts the microbial metabolism toward more energy-efficient pathways (aerobic respiration). Oxygen is crucial for the electron transport chain, a series of reactions that generate ATP, the main energy currency of cells. In aerobic respiration, each glucose molecule is fully oxidized through the citric acid cycle and the electron transport chain, yielding above 30 ATP molecules [25]. This high energy yield means microbes can meet their energy requirements with less glucose. In contrast, under anaerobic conditions, glucose is only partially oxidized, producing less ATP (about two ATP molecules per glucose molecule) [25]. As a result, microbes need to consume more glucose to generate the same amount of energy under anaerobic conditions. Therefore, by employing a BC bioreactor, we can enhance the microbial growth while reducing the resource utilization. This is due to the aerobic respiration pathway, which enables microbes to produce more energy from each molecule of glucose, leading to slower glucose consumption.

### 3.3. Protein Concentration

**Figure 4:**
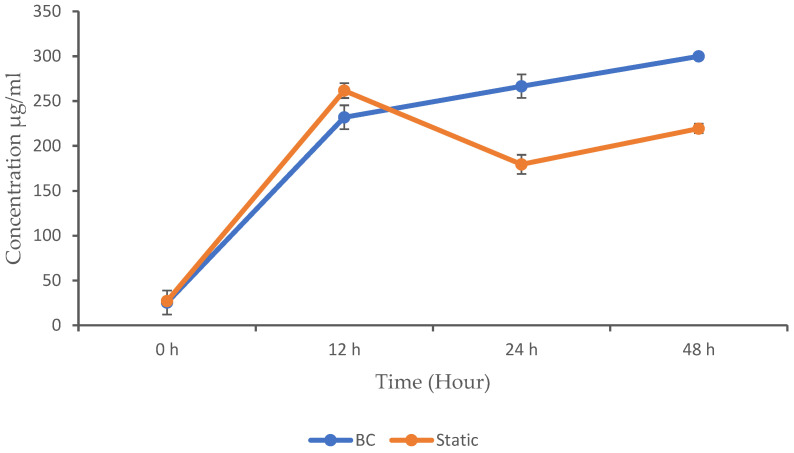
Microbial protein concentration in the BC and static conditions. The data are presented with mean values ± SD of n = 3 experiments.

Producing proteins with a BC bioreactor involves leveraging the unique characteristics, such as gas transfer and mixing, to optimize the growth and productivity of microorganisms. BC designs utilize gas bubbles to supply oxygen, mix the broth, and provide temperature regulation, potentially reducing capital costs and energy consumption compared to some conventional fermenters [19]. In this experiment, we compared the concentration of protein produced in a BC bioreactor with that produced during static fermentation. The results obtained are shown in Figure 4. Both methods started with relatively low protein concentrations, which indicates the beginning of the microbial activity. As the activity progressed, the microbial population increased, leading to an increase in the concentration of MPs. This is because proteins are crucial components of microbial cells and are produced in larger quantities as the microbial population expands. The static fermentation method shows a higher protein concentration (261.68 µg/mL) at 12 h compared to the BC (232 µg/mL). However, the *p*-value (<0.05) is not significant (but close), suggesting a potential difference that is not statistically confirmed. The protein concentration in the BC continued to increase and outperform the static fermentation, with a protein concentration of 266.62 µg/mL at 24 h and 299.90 µg/mL at 48 h, whereas the protein concentration in the static fermentation decreased to 179.43 µg/mL at 24 h and 219.44 µg/mL at 48 h.

The BC showed significantly higher protein concentrations at 24 and 48 h compared to the static method (*p* < 0.05), which indicate enhanced protein production in the BC bioreactor. No significant differences were observed at 0 and 12 h. Several studies have investigated protein production using BC bioreactors, with some identifying various factors that influence the growth and the yield of microbial proteins. Microbial growth and product formation in cultivation processes is closely linked to the availability of nutrients, especially carbon and nitrogen sources, which are essential for the microbial metabolism. In this study, we observed that all of the glucose in the static fermentation was completely consumed at 12 h, which explains the reduction in the growth and protein concentration that was experienced beyond this time point. The BC, however, maintained a steady glucose consumption rate as the growth and protein concentration increased.

Furthermore, our study indicates that static fermentation results in the higher protein production at 12 h, whereas the BC achieves its highest protein production at 48 h. This suggests that the cultivation method significantly impacts the timing of the protein yield. Interestingly, another study utilizing a type of BC bioreactor demonstrated the impact of the incubation time on the protein production. This study revealed a significant increase in protein production as the incubation time extended up to 10 h. However, beyond 20 h of cultivation, both the protein content and yield declined. The study found that the optimum incubation time for achieving the highest protein content was 8 h for *Aspergillus oryzae 3699*, with a protein content of 46.8%, and 10 h for *Rhizopus arrhizus 2062*, yielding 49.6% protein [26]. Tahir et al. [27] reported that the BC fermenter produced the highest protein yield, surpassing the stirred-tank bioreactor and demonstrating its efficacy in maximizing MP production. Another study also observed that the BC fermenter achieved the highest protein-rich biomass yield using *R. oligosporous*, outperforming the stirred-tank bioreactor [22].

Comparing these findings, it appears that the optimal incubation time for protein production differs between different organisms and cultivation methods. While the static fermentation in our study yielded the highest protein concentration at 12 h, the BC showed a longer incubation period, with higher protein production observed at 48 h. Overall, the BC seems more efficient than static fermentation throughout the study period. This could be due to the continuous mixing and aeration provided by the BC mechanism, which may enhance the cultivation process by ensuring optimal conditions for product formation. Additionally, the discrepancy between different studies may stem from variations in microbial strains, growth conditions, and reactor designs. Therefore, understanding these dynamics is crucial for optimizing protein production processes.

### 3.4. Trends in Organic Acid Production in the BC Reactor and Static Fermentation

**Figure 5:**
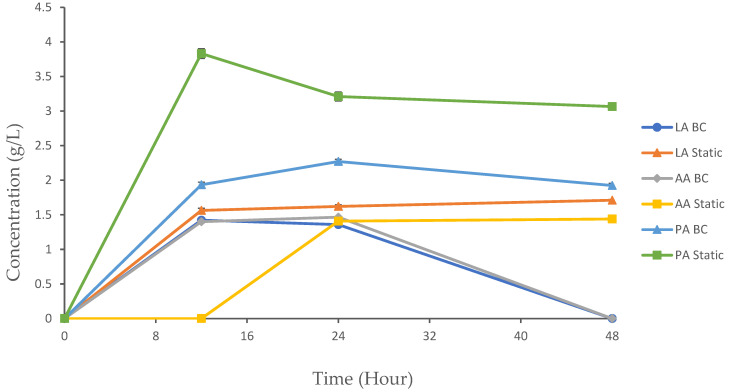
Organic acid production in the BC reactor and static fermentation. The data are presented with mean values ± SD of n = 3 experiments.

The experimental results provide valuable insights into the dynamics of organic acid production using the following two distinct methods: a BC bioreactor and static fermentation. These methods represent aerobic and anaerobic conditions, respectively, and their comparative analysis sheds light on how varying environmental conditions influence fermentation kinetics and product yield. Organic acids such as lactic acid (LA), acetic acid (AA), and propionic acid (PA) are important metabolic byproducts in microbial fermentation, with applications ranging from food preservation to pharmaceuticals. The dynamics of their production are critical to optimizing yields and achieving desired end-products. In this study, we monitored the concentration of metabolites produced over a 48 h period in both the BC and static reactors. LA, AA, and PA were detected using the HPLC system (Figure 5). All of the organic acids produced in both setups had an initial concentration of 0 g/L at 0 h.

#### 3.4.1. Lactic Acid

LA production is a key metabolic process during fermentation, where glucose is converted into lactic acid and adenosine triphosphate (ATP). The efficiency of this process can be influenced by various factors, including the fermentation method, the operation conditions, and the metabolic activity of the bacteria [28]. During the experiment, no significant differences were observed at 0 h. For the BC and static setups, the LA concentration significantly increased from 0 to 12 h, with 1.42 g/L and 1.56 g/L, respectively. Following this period, the concentration of LA in the BC chamber began to decline until it reached 0 g/L by 48 h. In contrast, the static fermentation method showed significantly higher LA concentrations at 12, 24, and 48 h compared to the BC reactor (*p* < 0.05).

Previous studies have highlighted the critical role of aeration in enhancing LA production, particularly for certain bacterial strains like *Bacillus* and *E. coli*. Qin et al. [29] noted that insufficient aeration limits LA production due to reduced biomass, whereas excessive aeration leads to the generation of undesirable byproducts. This underscores the delicate balance required in managing aeration levels to optimize the LA yield. Additionally, a study by Es et al. [30] reported that agitation and aeration within the bioreactor significantly influenced *Rhizopus oryzae* LA production [30]. These findings align with the general understanding of fermentation processes, where the efficiency of LA production can vary based on the method and conditions used [30].

In our study, we observed that the LA concentration significantly increased from 0 to 12 h in both the BC and static setups. However, following this period, the concentration of LA in the BC chamber began to decline until it reached 0 g/L at 48 h. This decline could be attributed to the microbes beginning to metabolize LA as a secondary energy source, even in the presence of glucose. The aeration in the BC system may have shifted the microbial metabolism toward oxidative pathways, leading to the consumption and depletion of LA over time. In contrast, the static setup demonstrated a more gradual rise in the LA concentration until 48 h. This difference can be attributed to the unique metabolic responses of microorganisms of choice to aeration [28]. While previous studies have successfully utilized a two-stage aeration control method to enhance LA production by adjusting aeration during the early stages of cultivation to boost biomass, and then reducing aeration to minimize byproduct formation [29,31], our findings indicate that continuous aeration in the BC led to a metabolic shift that decreased LA production over time.

Moreover, the relatively stable LA concentration observed in static fermentation suggests that anaerobic conditions favor continuous LA production. This is likely due to the absence of oxygen-induced byproduct formation and a more favorable metabolic environment for *K. oxytoca M5A1*. In contrast, under aerobic conditions in the BC bioreactor, microorganisms tend to shift towards oxidative metabolic pathways, which can result in the consumption of LA as an intermediate metabolite. These findings align with the work of Qin et al. [29], who reported that the excessive aeration in Bacillus sp. cultivation can lead to the formation of unwanted byproducts and reduce the LA yield. Our results further emphasize that a careful balance of aeration is essential for optimizing LA production, as excessive oxygenation may inadvertently cause microorganisms to metabolize LA, reducing its overall concentration.

#### 3.4.2. Acetic Acid

The BC and static fermentation methods showed significant differences in the AA concentration throughout the cultivation process. By 12 h, the BC displayed an increase in the AA concentration to 1.401 g/L, while the static method did not show any trait of AA production. The production of AA continued in the BC until 24 h, but hit a concentration of 0 g/L at 48 h. In the static condition, we initially detected a concentration of AA at 24 h (1.41 g/L), and it continued to increase until 48 h, reaching a final concentration of 1.44 g/L.

The BC showed significantly higher AA concentrations at 12 and 24 h compared to the static method (*p* < 0.05). At 48 h, the static method maintained higher AA levels, while the BC method showed no AA production. No significant differences were observed at 0 h. The observed differences in AA production between the BC and static methods are attributed to the fundamental differences in their operational environments. The BC supports early production of AA, which leads to a swift increase in the AA concentration. In a static environment, the presence of AA was observed after the complete consumption of glucose. After glucose is exhausted, microorganisms may shift towards utilizing alternative substrates or engage in alternative metabolic pathways to continue producing byproducts like AA. This metabolic adaptation is a common feature of microbial cultures during cultivation [32]. These findings indicate that the choice of cultivation method can significantly impact the rate and duration of AA formation as a byproduct. For instance, studies have shown that BCs often exhibit rapid initial production rates but may face challenges in maintaining productivity over time, whereas static fermentations tend to offer more stable, though slower, production profiles.

#### 3.4.3. Propionic Acid

PA, also known as propionate, is a carboxylic acid with commercial significance, primarily generated through microbial cultivation. Initially prevalent in the food sector, its utility has expanded to include applications in cosmetics, plastics manufacturing, and pharmaceuticals [33]. The production of propionate occurs through several metabolic routes, broadly categorized into the following three categories: fermentative pathways, biosynthetic pathways, and amino acid catabolic pathways [33]. In this study, PA was generated as a byproduct in the BC and static bioreactor systems. The BC showed a gradual increase in the PA concentration through 12 h (1.9 4 g/L) and 24 h (2.27 g/L). At 48 h, the concentration of PA decreased to 1.92 g/L. Static fermentation showed a more pronounced increase in the PA concentration at 12 h (3.83 g/L). However, the concentration began to fluctuate through 24 h and 48 h, with 3.21 g/L and 3.06 g/L, respectively. The static method showed significantly higher PA concentrations compared to the BC method at 12, 24, and 48 h (*p* < 0.05). No significant differences were observed at 0 h. The reduction in PA in the BC bioreactor at 48 h could be attributed to the microbes utilizing PA as an additional carbon or energy source alongside glucose. This is supported by the constant increase in both the growth rate and product yield observed at this stage. However, the production of PA under static conditions tends to be more productive than under BC conditions during cultivation. This is due to several key factors related to the metabolic preferences of the microorganisms involved, as well as the environmental conditions that favor the metabolism [34]. The fluctuations in PA production observed during static fermentation suggest possible oscillations in the metabolic activity. These variations could be influenced by shifts in microbial populations or the availability of substrates, leading to temporary pauses or slowdowns in the conversion of intermediates (such as pyruvate) to PA.

Many microorganisms, including those belonging to the genera *Propionibacterium*, *Selenomonas*, and *Clostridium*, preferentially produce PA under anaerobic conditions. These organisms have evolved to thrive in anaerobic conditions, where they can efficiently metabolize substrates such as carbohydrates, lipids, and proteins to produce PA along with other volatile fatty acids [35]. Anaerobic conditions allow these microorganisms to conserve energy by avoiding the oxidative phosphorylation pathway, which is energetically expensive. Instead, they rely on fermentation pathways to generate ATP. This makes anaerobic fermentation a more energy-efficient process for PA production [35]. A study reported that NADH and pyruvate play a pivotal role in controlling or limiting PA production. Pyruvate serves as a common intermediate in the metabolic pathway converting glycerol or glucose to propionate [33]. The availability of NADH determines whether the pathway will shift towards AA formation (due to insufficient reducing equivalents) or PA formation (with an adequate supply of NADH). Excessive pyruvate acts as an electron acceptor, leading to its oxidation to acetate instead of PA formation [34].

The observed differences in LA, AA, and PA production between the BC and static systems have important implications for optimizing bioprocesses in industrial applications. For instance, the stability of LA and PA production in static fermentation could be advantageous for processes that require consistent and prolonged acid production, such as food preservation, where a steady supply of organic acids over time is crucial for inhibiting microbial growth. On the other hand, BC systems, which show more dynamic changes in metabolite concentrations, may be better suited for processes that benefit from rapid metabolite turnover. In these systems, the continuous aeration and agitation promote faster metabolic cycles, leading to the quicker consumption and production of metabolites. This could be particularly useful in bioprocesses like bioconversion or bioremediation, where the rapid consumption and production of intermediates or byproducts is desirable. The ability to rapidly shift metabolic pathways in response to changing conditions makes BC systems ideal for applications requiring quick adaptation and fast production rates.

## 4. Conclusions

Our study highlights the impact of different cultivation methods—BC and static—on the growth, protein production, and metabolite profiles, using *K. oxytoca M5A1*. *K. oxytoca M5A1* was able to grow and produce MPs and metabolites in both systems, but with variations. The continuous mixing and aeration in the BC bioreactor led to a consistent increase in the microbial growth and protein concentration over 48 h, demonstrating the efficiency of this system. However, static fermentation exhibited a decline in protein production beyond 12 h, indicating that this method is less efficient for sustained growth and protein yield. The slower glucose consumption in the BC bioreactor, combined with the higher energy yield from aerobic respiration, supports the hypothesis that aeration enhances the metabolism, allowing microbes to utilize fewer resources while maintaining high growth rates. Our findings align with previous studies that show the benefits of BC bioreactors for improved cell mass growth and reduced resource utilization. Additionally, the choice of cultivation method significantly affects the production profiles of LA, AA, and PA. For processes that require the sustained production of acids, static fermentation may be the better option, while BC systems might be optimized for applications needing quicker metabolite production, provided that the balance between aeration and metabolite consumption is carefully managed. Future research could incorporate additional sampling points and explore the optimization of bioreactor configurations to maximize the MP yield in BC systems. Additionally, investigating the scalability of the process and the long-term stability of MP production under varying environmental conditions, such as the temperature, pH, oxygen levels, and nutrient availability, would provide valuable insights for industrial applications.

## Data Availability

Data are contained within the article.

## References

[1] Matassa S., Boon N., Pikaar I., Verstraete W. (2016). Microbial Protein: Future Sustainable Food Supply Route with Low Environmental Footprint. Microb. Biotechnol..

[2] Ciani M., Lippolis A., Fava F., Rodolfi L., Niccolai A., Tredici M.R. (2021). Microbes: Food for the Future. Foods.

[3] Kaur I., Sharma A.D. (2021). Bioreactor: Design, Functions and Fermentation Innovations. Res. Rev. Biotechnol. Biosci..

[4] Li G., Chen K., Wei Y., Zeng J., Yang Y., He F., Li H., Ouyang P. (2022). Mass Transfer, Gas Holdup, and Kinetic Models of Batch and Continuous Fermentation in a Novel Rectangular Dynamic Membrane Airlift Bioreactor. Engineering.

[5] Nourbakhsh F., Lotfalizadeh M., Badpeyma M., Shakeri A., Soheili V. (2022). From Plants to Antimicrobials: Natural Products against Bacterial Membranes. Phytother. Res..

[6] Sun J., Rutherford S.T., Silhavy T.J., Huang K.C. (2022). Physical Properties of the Bacterial Outer Membrane. Nat. Rev. Microbiol..

[7] Michenkova M., Taki S., Blosser M.C., Hwang H.J., Kowatz T., Moss F.J., Occhipinti R., Qin X., Sen S., Shinn E. (2021). Carbon Dioxide Transport across Membranes. Interface Focus.

[8] Zheng Y.-G., Chen X.-L., Wang Z. (2005). Microbial Biomass Production from Rice Straw Hydrolysate in Airlift Bioreactors. J. Biotechnol..

[9] Ale Enriquez F., Ahring B.K. (2023). Strategies to Overcome Mass Transfer Limitations of Hydrogen during Anaerobic Gaseous Fermentations: A Comprehensive Review. Bioresour. Technol..

[10] Waite C.J., Lindström Battle A., Bennett M.H., Carey M.R., Hong C.K., Kotta-Loizou I., Buck M., Schumacher J. (2021). Resource Allocation During the Transition to Diazotrophy in Klebsiella Oxytoca. Front. Microbiol..

[11] Aquino de Souza E., Rossi D.M., Záchia Ayub M.A. (2014). Bioconversion of Residual Glycerol from Biodiesel Synthesis into 1,3-Propanediol Using Immobilized Cells of Klebsiella Pneumoniae BLh-1. Renew. Energy.

[12] In S., Khunnonkwao P., Wong N., Phosiran C., Jantama S.S., Jantama K. (2020). Combining Metabolic Engineering and Evolutionary Adaptation in Klebsiella Oxytoca KMS004 to Significantly Improve Optically Pure D-(−)-Lactic Acid Yield and Specific Productivity in Low Nutrient Medium. Appl. Microbiol. Biotechnol..

[13] Behin J., Amiri P. (2023). A Review of Recent Advances in Airlift Reactors Technology with Emphasis on Environmental Remediation. J. Environ. Manag..

[14] Xu X., Zhang Y. (2024). Hydrodynamics and Mass Transfer in an Airlift Loop Reactor: Comparison between Using Two Kinds of Spargers. Processes.

[15] Kraakman N.J.R., Rocha-Rios J., van Loosdrecht M.C.M. (2011). Review of Mass Transfer Aspects for Biological Gas Treatment. Appl. Microbiol. Biotechnol..

[16] Prado Barragán L.A., Figueroa J.J.B., Rodríguez Durán L.V., Aguilar González C.N., Hennigs C. (2016). Fermentative Production Methods. Biotransformation of Agricultural Waste and By-Products.

[17] Ayodele T., Alarape K., Bello I.A., Tijani A., Musiliu L., Hammed A. (2024). Microbial Protein Production Using Lignocellulosic Biomass (Switchgrass) and Klebsiella Oxytoca M5A1—A Nitrogen Fixer. Sustainability.

[18] Spencer V.A., Davie J.R. (2002). Isolation of Proteins Cross-Linked to DNA by Cisplatin. The Protein Protocols Handbook.

[19] Uyar B., Ali M.D., Uyar G.E.O. (2024). Design Parameters Comparison of Bubble Column, Airlift and Stirred Tank Photobioreactors for Microalgae Production. Bioprocess Biosyst. Eng..

[20] Mohammadi K., Sohrabi Y., Heidari G., Khalesro S., Majidi M. (2012). Effective Factors on Biological Nitrogen Fixation. Afr. J. Agric. Res..

[21] Hosseini M., Shojaosadati S.A., Towfighi J. (2003). Application of a Bubble-Column Reactor for the Production of a Single-Cell Protein from Cheese Whey. Ind. Eng. Chem. Res..

[22] Nadeem H. (2021). Conversion of Potato Peels into Single Cell Protein: Potato Peels into Single Cell Protein. Futur. Biotechnol..

[23] Yen H.-W., Chang J.-T. (2015). Growth of Oleaginous *Rhodotorula glutinis* in an Internal-Loop Airlift Bioreactor by Using Lignocellulosic Biomass Hydrolysate as the Carbon Source. J. Biosci. Bioeng..

[24] Rossi M.J., Nascimento F.X., Giachini A.J., Oliveira V.L., Furigo A. (2017). Transfer and Consumption of Oxygen during the Cultivation of the Ectomycorrhizal Fungus Rhizopogon Nigrescens in an Airlift Bioreactor. Appl. Microbiol. Biotechnol..

[25] Melkonian E.A., Schury M.P. (2024). Biochemistry, Anaerobic Glycolysis. StatPearls.

[26] Jin B., Yu Q., van Leeuwen J. (2001). A Bioprocessing Mode for Simultaneous Fungal Biomass Protein Production and Wastewater Treatment Using an External Air-Lift Bioreactor. J. Chem. Technol. Biotechnol..

[27] Tahir F., Hussain A., Shamaiz S., Uzair M., Zubair F., Saeed A., Iqbal S., Sikandar M., Nauman M. (2023). Animal Science Journal From Scraps to Protein Powerhouse: Transforming Potato Peels into Single Cell Protein. Anim. Sci. J..

[28] Abdel-Rahman M.A., Tashiro Y., Sonomoto K. (2013). Recent Advances in Lactic Acid Production by Microbial Fermentation Processes. Biotechnol. Adv..

[29] Qin J., Wang X., Zheng Z., Ma C., Tang H., Xu P. (2010). Production of L-Lactic Acid by a Thermophilic Bacillus Mutant Using Sodium Hydroxide as Neutralizing Agent. Bioresour. Technol..

[30] Eş I., Mousavi Khaneghah A., Barba F.J., Saraiva J.A., Sant’Ana A.S., Hashemi S.M.B. (2018). Recent Advancements in Lactic Acid Production—A Review. Food Res. Int..

[31] Zhu Y., Eiteman M.A., DeWitt K., Altman E. (2007). Homolactate Fermentation by Metabolically Engineered *Escherichia coli* Strains. Appl. Environ. Microbiol..

[32] Huo G., Foulquié-Moreno M.R., Thevelein J.M. (2022). Development of an Industrial Yeast Strain for Efficient Production of 2,3-Butanediol. Microb. Cell Factories.

[33] Gonzalez-Garcia R.A., McCubbin T., Navone L., Stowers C., Nielsen L.K., Marcellin E. (2017). Microbial Propionic Acid Production. Fermentation.

[34] Liu Y., Zhang Y.-G., Zhang R.-B., Zhang F., Zhu J. (2011). Glycerol/Glucose Co-Fermentation: One More Proficient Process to Produce Propionic Acid by Propionibacterium Acidipropionici. Curr. Microbiol..

[35] Ranaei V., Pilevar Z., Khaneghah A.M., Hosseini H. (2020). Propionic Acid: Method of Production, Current State and Perspectives. Food Technol. Biotechnol..

